# A Freidsonian Analysis of The Professional trajectories of Public Health Nursing Specialists in Minas Gerais, Brazil, 1988–1992

**DOI:** 10.1590/1980-220X-REEUSP-2024-0191en

**Published:** 2024-11-08

**Authors:** Isabella Lara Maia de Carvalho, Pacita Geovana Gama de Sousa Aperibense, Alice Gomes Frugoli, Maria Angélica de Almeida Peres, Sagrario Gómez-Cantarino, Fernanda Batista Oliveira Santos

**Affiliations:** 1 Universidade Federal de Minas Gerais, Escola de Enfermagem, Graduação em Enfermagem, Belo Horizonte, MG, Brazil.; 2 Universidade Federal do Rio de Janeiro, Departamento de Enfermagem Fundamental, Macaé, RJ, Brazil.; 3 Universidade Federal de Minas Gerais, Escola de Enfermagem, Programa de Pós-graduação em Enfermagem, Belo Horizonte, MG, Brazil.; 4 Universidade Federal do Rio de Janeiro, Escola de Enfermagem Anna Nery, Departamento de Enfermagem Fundamental, Rio de Janeiro, RJ, Brazil.; 5 Universidad de Castilla-La Mancha, Facultad de Fisioterapia y Enfermería del Campus de Toledo, Departamento de Enfermería, Fisioterapia y Terapia Ocupacional, Toledo, Espanha.; 6 Universidade Federal de Minas Gerais, Escola de Enfermagem, Departamento de Enfermagem Básica, Belo Horizonte, MG, Brazil.

**Keywords:** History of Nursing, Education, Nursing, Graduate, Specialization, Health Occupations, Historia de la Enfermería, Educación de Postgrado en Enfermería, Especialización, Empleos en Salud

## Abstract

**Objective:**

To analyze the contributions of the Public Health Nursing Specialization Course (1988–1992) of *Universidade Federal de Minas Gerais* in improving the expertise and professional status of its graduates.

**Method:**

Historical and social study in the history of nursing and expertise. The sources included 20 registration forms, enrollment documentation of graduates, and three course documents collected in the Memory Center of *Escola de Enfermagem da Universidade Federal de Minas Gerais*, combined with Lattes/CNPq curricula and analyzed through Eliot Freidson's Sociology of Professions.

**Results:**

The specialists were selected through a strategy of construction of knowledge for socio-sanitary transformation. The specialization provided support for the professional career of graduates in administrative leadership, management, and visibility positions in public health; the group's profile was focused on academia, which creates its own knowledge.

**Conclusion:**

The specialization provided its graduates the opportunity to exercise professional autonomy, elevating the state of affairs and contributing to the professionalization of public health nursing.

## RESUMEN

**Objetivo:**

Analizar las contribuciones del Curso de Especialización en Enfermería de Salud Pública (1988–1992) de la *Universidade Federal de Minas Gerais* en el fortalecimiento de la especialización y del estatus profesional de sus egresados.

**Método:**

Estudio histórico-social sobre la historia de la enfermería y de la especialización. Las fuentes incluyeron 20 fichas de inscripción, documentos de matrícula de los egresados y tres documentos del curso obtenidos en el Centro de Memoria de la *Escola de Enfermagem da Universidade Federal de Minas Gerais*, combinados con los currículos Lattes/CNPq y analizados a partir de la Sociología de las Profesiones de Eliot Freidson.

**Resultados:**

Los especialistas fueron seleccionados mediante una estrategia de construcción de conocimiento para la transformación sociosanitaria. La especialización apoyó el desarrollo de la carrera profesional de los egresados en roles de liderazgo administrativo, gestión y visibilidad en salud pública; el perfil del grupo estuvo orientado hacia el ámbito académico, generador de su propio conocimiento.

**Conclusión:**

La especialización brindó a los egresados la oportunidad de ejercer la autonomía profesional, mejorando el estado de la situación y contribuyendo a la profesionalización de la enfermería en salud pública.

## INTRODUCTION

The Specialization Course in Public Health Nursing (*Curso de Especialização em Enfermagem de Saúde Pública* – CEESP) offered between 1988 and 1992 was one of the first postgraduate nursing initiatives in the state of Minas Gerais, Brazil. It used to be taught at *Escola de Enfermagem da Universidade Federal de Minas Gerais* (EEUFMG), an institution created in 1933, as part of the strategies for state investment in public health in Brazil^(1)^.

In the context of early 20th century public health issues, the Brazilian government focused heavily on the qualified training of nurses as a strategy to reformulate the country’s health care model. Thus, the history of Brazilian nursing has been based since its beginnings on public health and the paradigm of modernity. They have developed together, consolidating the professionalism that nursing holds today before society and the State^(2)^.

Throughout the second half of the 20th century, Brazilian public health underwent significant reconfigurations driven by the struggle for civil rights. The 1986 Health Reform expanded the concept of health. The necessary changes transcended the limits of an administrative and financial reform. The Constitution created the Unified Health System (*Sistema Único de Saúde* – SUS) and assigned the Brazilian Ministry of Health (MH) the role of fostering and encouraging scientific and technological development and innovation in the health field, as recommended by article 200 of the Federal Constitution^(3,4)^.

Historically, Brazilian nursing schools have been reconfigured in response to changes in the sociopolitical context. These changes were fundamental to the process of professionalizing nursing in Brazil. The reformulations of the national health system in the 1980s also had an impact on the training offered by EEUFMG. The institution was a protagonist in extramural public health actions in Minas Gerais, such as vaccination campaigns, emergency response to floods, and the provision of free nursing services in several municipalities in the state. It was a pioneer in the professionalization of nursing in Minas Gerais, implementing a teaching method that also valued public health nursing, with important contributions to the national and international health scenario^(5,6)^.

From the perspective of Eliot Freidson’s Sociology of Professions, which was designed for the medical profession, two elements stand out in professionalization: expertise and professional status. Expertise is the specific professional knowledge provided by conventional expert education in professional schools and is shaped by regulated and formal training. Professional status represents the technical and legal authority within the division of labor of an occupation and reflects the profession’s significance for society^(7)^.

In nursing as a professional field, Freidson’s theoretical framework gained prominence in epistemological studies, especially those of a historical nature(^1,2,6,8,9,10^). Researchers identified Freidson’s professionalization factors in nursing work, concluding that nurses are autonomous professionals with their own professional practice regulations(^2^). When we apply this analysis to nursing, establishing this professional and scientific field as a space to respond to social health needs, professional qualification and self-production of knowledge have proven to be fundamental(^2,5^). Nurses should integrate into society and make their process of knowledge construction and practice visible and occupy important positions to be valued by their peers and by those who benefit from their work.

From this perspective, although CEESP is recognized in the literature, the implications of the public health education provided in this postgraduate nursing course in Minas Gerais have not been elucidated^(6)^. Furthermore, the characterization of the subjects involved and the strategies adopted in the Brazilian Health Reform have been so far insufficiently explored in studies on this phenomenon. Therefore, visibility should be given to the historical undertakings of nursing in Brazilian public health, as well as professional training, to take on such initiatives, highlighting the performance of this category in the health sector at the national level(^11,12,13^).

It was assumed that nurses who attended CEESP at UFMG and dedicated themselves to building a professional career based on the development of their own field-specific knowledge occupied prominent positions, achieving professional status. Therefore, this study aimed to analyze the contributions of the Specialization Course in Public Health Nursing (1988–1992) at *Universidade Federal de Minas Gerais* in enhancing the expertise and professional status of its graduates.

## METHOD

### Study Design

This is study in the field of History, with a historical and social dimension, covering the domains of the history of expertise and the history of nursing, with a documentary/textual history approach^(14,15)^. A study of the graduates’ records was carried out, identifying the positions held by graduates after completing the Specialization Course in Public Health Nursing.

### Location

The study setting was *Escola de Enfermagem da Universidade Federal de Minas Gerais*, located in Belo Horizonte, the capital of the state of Minas Gerais, Brazil. The time frame spanned from 1988, when the first class entered CEESP, to 1992, when the last class graduated, according to the available records.

### Selection Criteria

The Memory Center of Escola de Enfermagem da UFMG (CEMENF) has a documentary collection on the nursing specialization courses offered by the institution. The document corpus (population) of the CEESP collection consists of: CEESP Creation Plan (1987), CEESP 1988–1989 Activity Report (1989), 84 course registration forms and enrollment and identification documentation of CEESP graduates who completed the course between 1988 and 1992, including enrollment application, proof of completion of undergraduate course, high school and higher education transcripts, curriculum, certificate of leave and statement of recommendation, natural persons register, identity card, proof of voting, and certificate of military status.

The inclusion criteria for direct sources were mention or open discussion of this specialization course, registration forms, and identification documentation of CEESP graduates who completed their studies between 1988 and 1992. The exclusion criteria were illegible and/or damaged forms with compromised readability and understanding. In this first stage, 83 forms were selected.

In addition, to compile data on the professional trajectory of graduates, searches were conducted on the Lattes Platform, maintained by the Brazilian National Council for Scientific and Technological Development (*Conselho Nacional de Desenvolvimento Científico e Tecnológico* - CNPq). This platform was chosen because it is the main Brazilian virtual system for scientific curricula and has open access, thus allowing confirmation of graduate identity based on the triangulation of sources. The curricula of CEESP graduates from 1988 to 1992 were included. Curricula that did not provide enough information to map the professional trajectory of graduates were excluded, in order to identify the influence of the course on acquiring expertise and enhancing the status of this group. As a result, in this second stage, twenty curricula comprised the final sample.

### Data Collection

Data collection took place between December 2023 and January 2024, in the CEMENF collection and on the Lattes Platform, applying the inclusion and exclusion criteria. The documents were digitized and saved on an online data storage platform, cataloged, compiled into an Excel® spreadsheet and, later, were made available by the museum as a public domain documentary collection through the AtoM^®^ software (Access to Memory – an archival description application, based on the standards of the International Council on Archives).

Lattes/CNPq Platform was then searched for CEESP graduates. Since the CEMENF documentation allowed their nominal identification, each of them was searched for, and their full names were entered into the curriculum search engine on the website https://buscatextual.cnpq.br/buscatextual/busca.do?metodo=apresentar.

### Data Analysis and Treatment

After the heuristic or initial reading and collection of all CEESP documents, the search began for documents of interest for hermeneutics in terms of knowing to what extent the information provided by them answered the questions initially raised. To this end, internal and external criticism of the documents was carried out, verifying their validity and coherence from the source itself, as well as their veracity regarding external factors and the socio-sanitary context of the 1980s and 1990s. A document file was used as an instrument to extract information regarding CEESP graduates in this phase. It contained the following questions: “What are the contributions of CEESP (1988–1992) at UFMG to its graduates’ trajectories? Did CEESP enhance the expertise and professional status of its graduates?”

The findings were then analyzed considering Eliot Freidson’s theoretical framework of health professionalization and its interpretations for the professional field of nursing^(2,7)^.

### Ethical Aspects

The research used documents from a public archive and a virtual platform with open internet access. In addition, to guarantee the privacy of the information of the graduates and to prevent their identification, the data were anonymized, with no conflict of interest and no need for an Ethics Committee opinion, as provided for in Resolutions 466/2012 and 510/2016 of the Brazilian National Health Council (*Conselho Nacional de Saúde* - CNS).

## RESULTS

The document titled *Plano de Criação do CEESP* (CEESP Creation Plan) (1987) shows that the project for the Specialization Course in Public Health Nursing was designed to deepen nursing-specific knowledge and foster the development of professional skills in outpatient care. The objective of the course was to train nurses to provide services and to systematically study nursing work in primary care. Furthermore, the main document for the creation of CEESP proposes that this specialization course should support and pave the way for the creation of the Master’s Program in Public Health Nursing. The master’s degree in Nursing was started at EEUFMG in 1994, with strong involvement from public health nursing professors^(5)^.

The document *Relatório de Atividades 1988–1989* (Activity Report 1988–1989) (1989) described the course’s development within a nationwide teacher training program initiated by the Nursing Schools of three Brazilian universities: *Universidade Federal da Bahia*, *Universidade de São Paulo*, and *Universidade Federal de Minas Gerais*. Remarkably, CEESP was the first course to be implemented based on a national decision to specialize primary care nurses, called “basic health care” by *Movimento Sanitário Brasileiro* (Brazilian Health Movement) at that time^(16)^, aiming to consolidate the Health Reform. It was promoted, through partnerships and agreements, with the technical and financial support of the Brazilian National Institute of Medical Assistance of Social Security (*Instituto Nacional de Assistência Médica da Previdência Social* – INAMPS), the Pan American Health Organization (PAHO), the Brazilian Ministry of Health, State Department of Health of Minas Gerais (*Secretaria de Estado da Saúde de Minas Gerais* – SES/MG), and the Municipal Department of Health of Belo Horizonte (*Secretaria Municipal de Saúde de Belo Horizonte* – SMS/BH). Thus, EEUFMG took on not only the role of qualifying nurses involved in primary care, but also the role of forming reference centers for other Nursing Schools in Brazil through public and completely free education, honoring its responsibility by returning the government’s investment to society.

The program’s curriculum included subjects on nursing training for the role of supervisor-instructor, the control of communicable diseases, women’s and children’s health, urban and rural men’s health, pedagogical training, and the administration of methodological services and processes. The total workload was 960 hours, distributed into two academic semesters at the University, with practical activities carried out in four Health Centers in Belo Horizonte, defined as fixed sites for all courses, and in variable sites depending on the nature and specificity of each course. For comparison purposes, in the 1980s and 1990s in Brazil the workload of specialization programs varied with education centers and their guidelines. However, as of 1999 Resolution CNE/CES nº 3, the minimum workload for *lato sensu* postgraduate programs became 360 hours^(17)^, which shows the academic rigor and depth of the training offered by CEESP, preparing distinguished professionals for the job market.

Course enrollment required a duly filled application form, proof of completion of the Nursing and Obstetrics undergraduate course, academic transcript, curriculum, proof of voting and certificate of military status, and a statement from the employer regarding the importance of the course for the candidates’ work and the willingness to allow them to take the course. The selection process was carried out with the agreement among supporting institutions and priority was given to nurses indicated by them, with the following distribution of the thirty spots for each semester of the program: ten spots for nurses indicated by INAMPS; ten spots for nurses indicated by SES/MG; five spots for nurses indicated by SMS/BH; five spots for nurses selected by EEUFMG, with priority given to professors of the undergraduate nursing course. Any remaining spots would be made available for nurses in general and, if the number of candidates exceeded the number of spots, interviews and curriculum analyses would be conducted by an examining board set up for this purpose.

**Figure 1:**
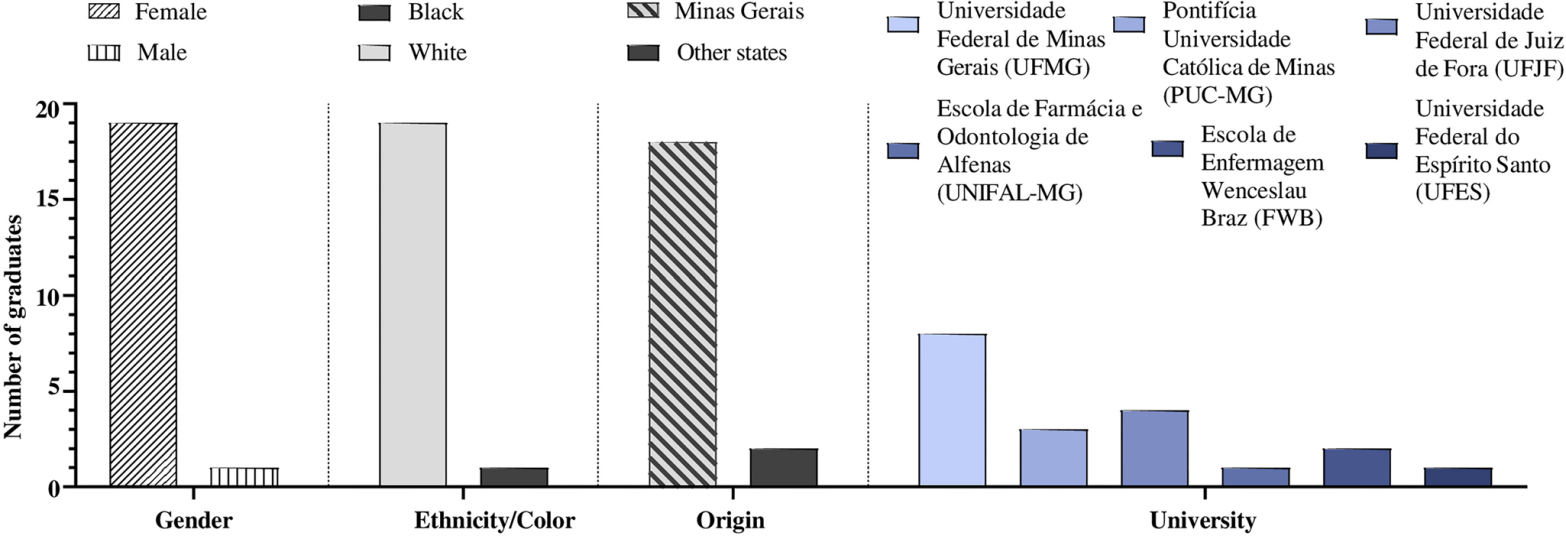
Frequency of ethnicity/color, gender, origin, and higher education institutions of graduates.

According to the documents, between 1988 and 1992 at least 83 students were awarded the title of Specialist in Public Health Nursing by the program. However, access to direct sources from CEMENF allowed the characterization of the professional careers of twenty CEESP graduates based on admission/identification forms and Lattes curricula. The graduates were predominantly women (95%) with an average age of 27 years and self-declared white (Figure 1). It is important to highlight the presence of one black female graduate and two male graduates. The students were predominantly born in the state of Minas Gerais, in fifteen municipalities. Graduates from the states of Rio de Janeiro and Rio Grande do Sul must also be emphasized.

Most students had graduated from universities in Minas Gerais, located in the cities of Belo Horizonte, Juiz de Fora, Alfenas and Itajubá; the most frequent ones were UFMG, *Pontifícia Universidade Católica de Minas Gerais* (PUC-MG) and *Universidade Federal de Juiz de Fora* (UFJF) (Figure 1).

The sources also showed that almost all of the nurses who enrolled in the course had worked in the public health sector. Among the enrollment documentation presented by former students to enroll in the course, employer statements served as letters of recommendation to attest to the relevance of the course in their professional training; these were written by superintendents of the positions previously held by the students.

**Figure 2:**
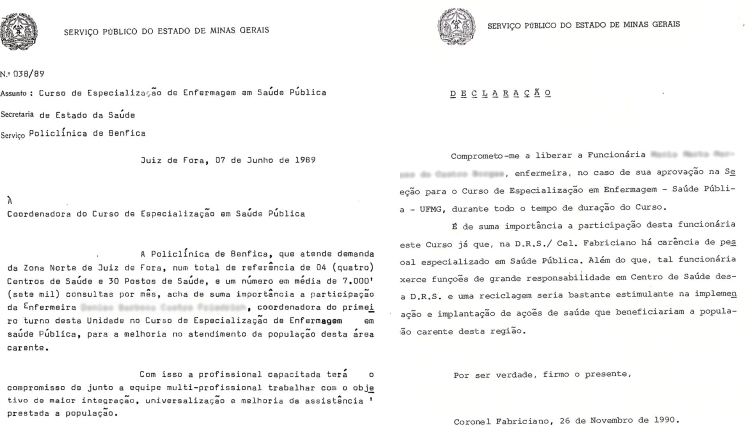
Student recommendation letters. Source: Enrollment documentation for the Specialization Course in Public Health Nursing 1989–1990, collection of the Memory Center of *Escola de Enfermagem da Universidade Federal de Minas Gerais*.

The eleven recommendations, all of which are available in the CEMENF collection, were authored by state agencies (Figure 2). The administrative support provided by the state network units – where some of these graduates worked – for professional qualification must be highlighted, as this shows the importance of this activity for “improving the assistance provided to the population” and for management activities.

Although students joined the course by referral, it is worth noting that the candidates had taken other postgraduate courses, namely Development in Hospital Management, Human Resources Management, Management of Hospital Units, Strengthening of Immunization Actions in Municipal Territories, Management and Administration Services of the Nursing Work Process, Qualification in Public Health Nursing, and Specialization in Public Health, in addition to CEESP/UFMG’s course. Furthermore, six of the twenty graduates had an average of two or more postgraduate courses.

The analysis of the graduates’ curricula indicates that these professionals stood out in their work in the public sector from six months to one year after finishing CEESP. This was true both for those who remained in the state of Minas Gerais (fourteen of the twenty graduates) and for those who returned to their respective hometowns to collaborate with the local health sector (six of the twenty graduates).

Regarding positions held while taking the course, all twenty graduates continued to be public servants and worked in different health service institutions, including Municipal Health Departments and the State Health Department of Minas Gerais, Health Centers, hospitals, polyclinics, outpatient clinics, Medical Care Posts, Basic Health Units, Regional Health Coordination Office, and public undergraduate and postgraduate Nursing courses in Minas Gerais.

The positions held by graduates in the public sector that stood out the most after completing the Specialization were Management of a Basic Health Unit, Management and Administration of the outpatient sector of *Hospital das Clínicas da UFMG*, Nursing Supervision, Management of the Metropolitan Regional Health Office, Coordination and Supervision of Multiple Vaccinations, Management of the Department of Maternal and Child Nursing and Public Health of the School of Nursing of UFMG, Specialist in Health Management Policy, Supervising Instructor and Professor at EEUFMG. Furthermore, among these graduates, ten had double shifts; two of them commuted between Belo Horizonte and cities in the metropolitan region.

Professional activity varied among the following dimensions of the nursing work process: care (20%), management (37%), research (3%), and education (40%). Only one graduate worked in the private sector by teaching at a private higher nursing education institution in Belo Horizonte. Regarding the advancement of their professional qualifications after CEESP, 95% had more than one degree and completed at least one type of course among the three: Specialization (33), Master’s (13), or Doctorate (6).

Sixteen of the graduates became specialists in their respective Master’s and/or Doctorate study subjects: Management of Basic Health Units, Planning and Administration of Health Systems, Occupational Nursing, Ergonomics, Emergency and Mobile Pre-hospital Care, Family Health, Professional Education in the Health Area, Microregional Health Management, Higher Education Teaching, Activation of Change Processes, Stomatherapy, Public Health Nursing for the Unified Health System, Health Surveillance, Strategic Health Planning and Management, Gerontological and Geriatric Nursing, Epidemiology in Health Services, Micropolitics of Health Management and Work, and Worker Health Epidemiology.

Twelve of these graduates earned Master’s degrees in the area of Health Sciences and in the field of Nursing. The sub-fields varied between Public Health Nursing, Epidemiology, Family Health and Primary Care, Health, and Social Services. The specialties included Public Health, Health Care of Human Populations, Environment and Sustainability, Economic Policy and General Public Administration, Social Vulnerability, Women’s and Newborn Health, Health Planning, and Evaluation of Information Systems. One graduate earned a Master’s degree in the area of Applied Social Sciences, in the area of Administration and in the sub-area of Public Administration, in the specialty Defense and Social Security. Ten of the twelve graduates took other specialization courses prior to their Master’s degree.

Of those mentioned above, five graduates received their PhDs in the area of Health Sciences and in the area of Nursing. The sub-area consisted of Public Health and Contagious Disease Nursing, and the specialties included Family Health and Primary Care, Organization Process in Health Services, Chagas Disease, Sexually Transmitted Infections in Adolescence, and Public Administration Services in the Social Security Sphere. One graduate was awarded a PhD in the area of Biological Sciences, the sub-area of Parasitology and Epidemiology, and the specialty Dengue Fever. All six of these graduates completed a Master’s degree before starting their PhD.

Regarding the current positions or the last ones among the graduates, 18 are statutory public servants or working under the Consolidation of Labor Laws (*Consolidação das Leis do Trabalho* - CLT), linked to the following health services: Municipal Health Departments and State Health Department of Minas Gerais, hospitals, Regional Health Coordinators, Department of Worker Health Care, Hospital Foundation of the State of Minas Gerais, Ministry of Health, Mobile Emergency Care Service (*Serviço de Atendimento Móvel de Urgência* - SAMU), Reference Center for Workers’ Health and Nursing and Medicine undergraduate and postgraduate courses in universities in Southeast Brazil.

Regarding the levels of the positions held by CEESP graduates, high-level positions stand out, including Professor and Researcher of Nursing at UFJF and UFMG, Coordinator of Nursing at *Hospital das Clínicas da UFMG*, Coordinator of Epidemiology, Director of Health Surveillance, Secretary of Epidemiological Surveillance, General Coordinator of SAMU and Work Management Superintendent. In addition, voluntary connections with the Brazilian Nursing Association (*Associação Brasileira de Enfermagem* - ABEn Nacional) and participation in the Editorial Board of *Revista Mineira de Enfermagem* (REME) were identified.

The type of activity performed varied among the four dimensions of nursing work: care (3.7%), management (37.04%), research (18.52%), and teaching (40.74%). Five graduates worked double shifts, with two continuing to work in teaching and research activities in the private sector. These professionals worked at universities in Minas Gerais, offering Nursing and Medicine undergraduate and postgraduate courses. Their positions were Professor and Researcher and Coordinator of the Nursing Course.

## DISCUSSION

The syllabus as described in the course project explicitly highlights their attentiveness to transmitting knowledge in public health, with the purpose of recovering technical competence, forging the knowledge specific to the profession and qualifying professionals as references for the Health Reform^(6)^. Furthermore, with the implementation of the principles of SUS and primary health care, it became necessary to train nurses to be capable of intervening politically and administratively in the process of reorganizing local health services^(18)^, which leads us to infer that the disciplines of pedagogical training and administration of services and methodological processes comprised the curriculum with a view to developing these skills and competencies.

Regarding the socioeconomic profile of the graduates, there is a noticeable socially spread historical influence^(19)^, perpetuated by a professional identity strongly related to religion and the Nightingale standard, leading to a majority of young white women in higher education nursing courses. However, a specialist degree being awarded to one black woman and two men by CEESP is a remarkable fact, as it reaffirms the effort – promoted by EEUFMG since its creation – to overcome elitist training paradigms and to break with segregation and inequality in labor relations. This school was a pioneer in training a black nurse in its first decade of operation^(1)^.

**Figure 3:**
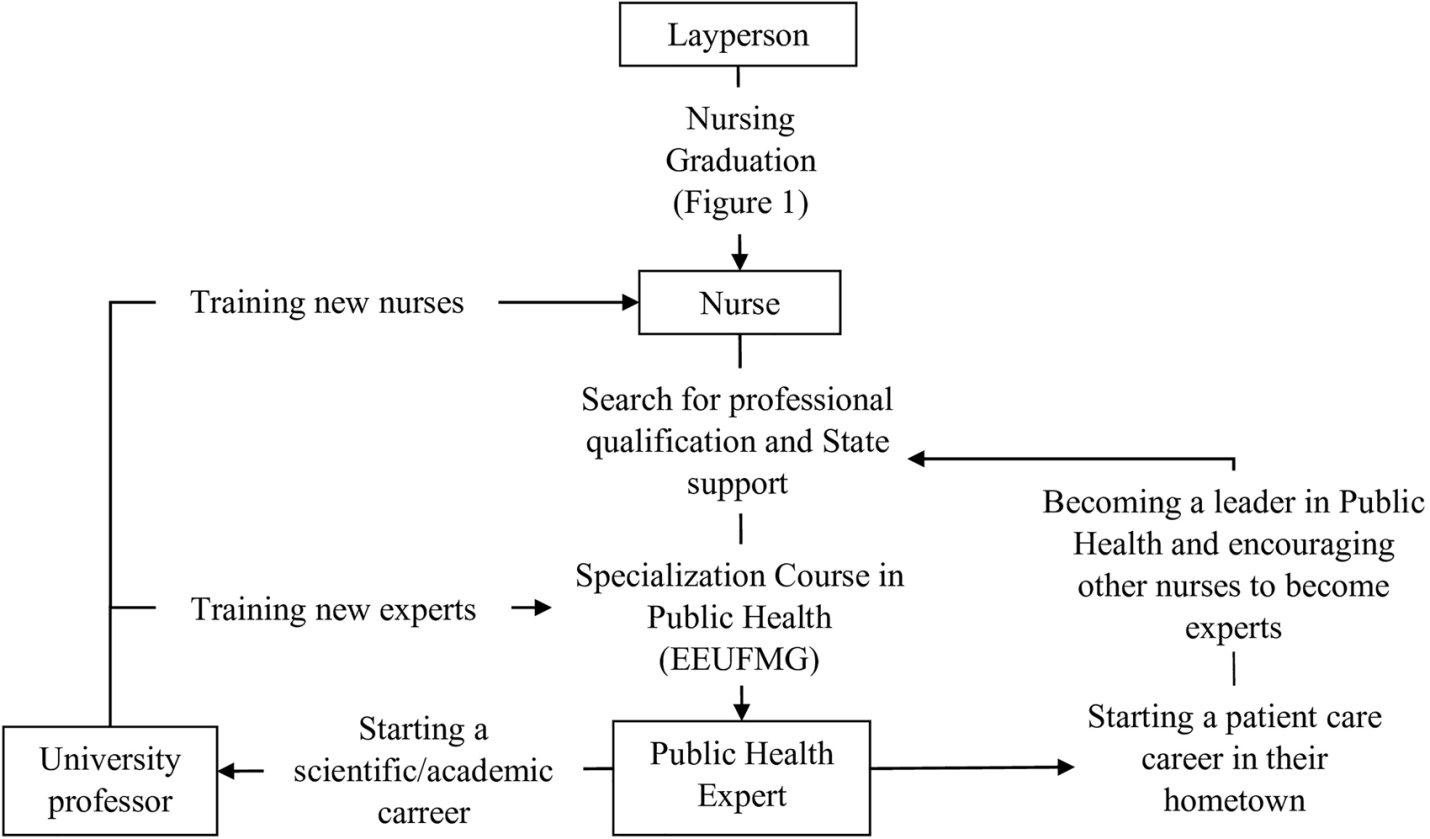
Professional flow of graduates from the Specialization Course in Public Health Nursing.

The analysis of the professional trajectories of the graduates allowed us to define a professional flow (Figure 3). As most were born in Minas Gerais, they received their academic training in its capital, Belo Horizonte; upon completion, they returned to their respective hometowns and, after completing their postgraduate studies, being awarded the title of specialist, they assumed leadership positions in public health in their hometowns and/or became nursing professors. This contrasts with the historical reality of the early days of specialization for nursing professionals in Minas Gerais. It is worth noting that the flow in Figure 3 refers to the CEESP graduates characterized in Figure 1.

It is known that the first professors to receive specialist, master’s and doctorate degrees in Minas Gerais had to obtain qualifications outside the state and outside Brazil. However, after completing these qualifications, they returned to EEUFMG to train new nurses and promote the creation of specialization courses in Minas Gerais, as well as to lead actions to professionalize nursing in this state and nationwide(^1,5,6^).

**Figure 4:**
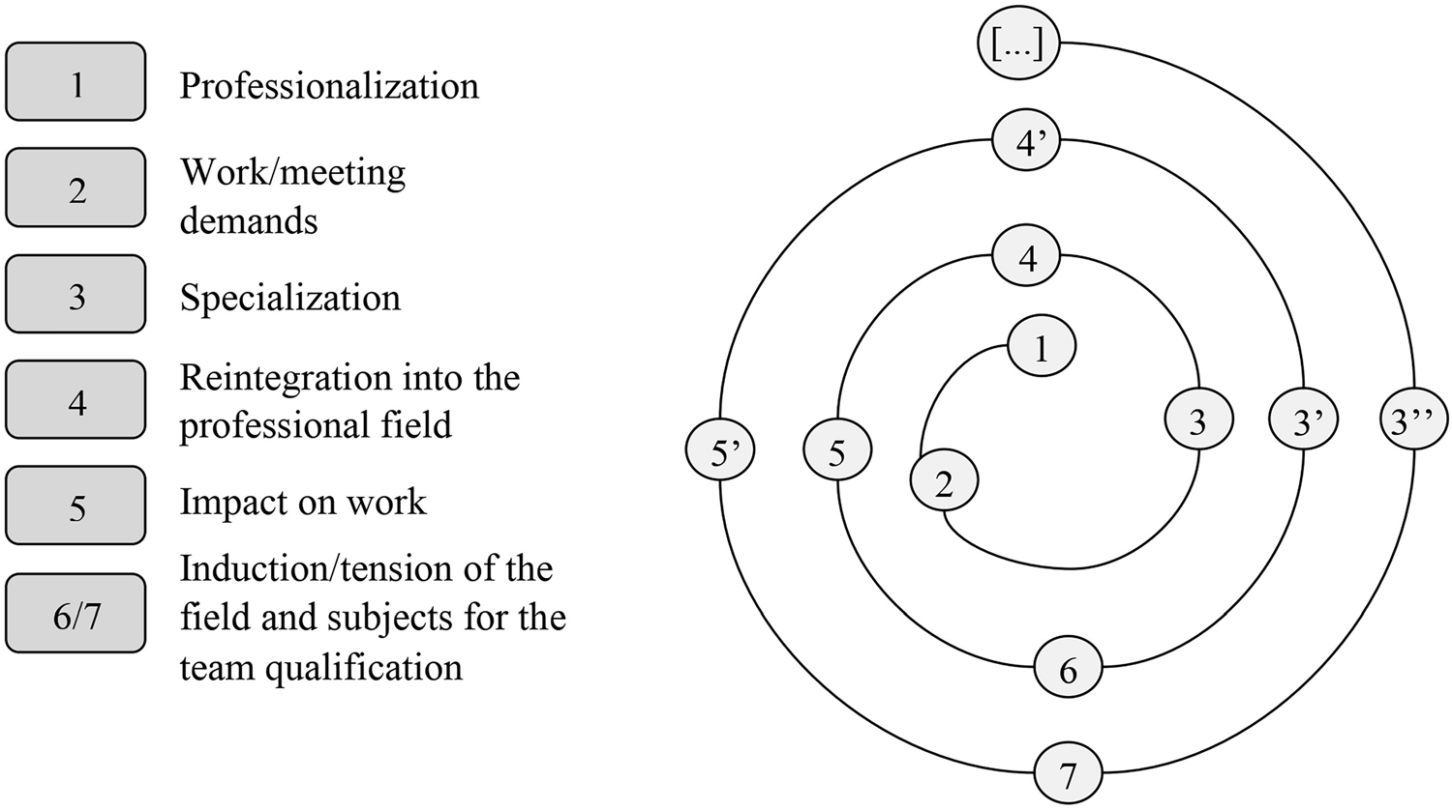
Cycle of professionalization of Public Health Nursing in Minas Gerais in the late 1990s.

A professionalization cycle (Figure 4) is thus highlighted; when occurring in nursing in Minas Gerais, it reflects the archetype of the first nurses trained at the Anna Nery Nursing School, pointing to the social commitment that these pioneers had with their training and practice environments^(5)^. In addition, it can be inferred that citizens born in Minas Gerais municipalities, with public health challenges, aimed to become professionals and individually acquire expertise^(7)^ in this area. After specialization, they began to lead local public health services, training the professionals who worked there and motivating them to specialize, as shown in Figure 4.

The field of professionalization follows a conformation trend: the course itself is strained by the movement towards professionalization and meeting demands (1 and 2 in Figure 4), incorporating socio- sanitary needs as instruments for self-transformation (3 in Figure 4), the field in which it operates (4 in Figure 4), and the subjects involved (5 in Figure 4). These, in turn, also begin to reposition themselves in this field (6 in Figure 4) at different points, causing impacts on their work (3’ in Figure 4), which will be unique (4’ in Figure 4) and in tune with new social needs; thus, successively, professionals with expertise influence other professionals, including recent graduates, maintaining the spiral of knowledge in a constant, progressive development (3’’ and [...] in Figure 4).

Regarding the process of professionalization of public health nurses in Minas Gerais, 55% of the specialist qualifications granted by CEESP were directly supported by the health services that employed the prospective students through a letter of recommendation. This demonstrates that CEESP held social value before the State, which invested in the qualification of nurses as the profession that holds expertise in public health, thus protecting their space of autonomy and body of knowledge in this area^(2,7)^. Therefore, authority is noted in the work of nurses in public health in Minas Gerais.

It is also important to note that the nurses themselves, after receiving their degrees, took on teaching and/or leadership roles, fostering training in the services that recommended them, highlighting their mastery of their body of knowledge^(7)^ and scope of work in public health, supporting the process of internalizing nursing professionalization in the state of Minas Gerais. Furthermore, the expertise of public health nurses trained by CEESP was evidenced by the observation of the theoretical requirements of professional training postulated by Freidson^(7)^, which are the acquisition of specific knowledge through training in a professional school subject to formal regulations, such as EEUFMG.

Furthermore, according to Freidson, expertise is expressed in the professional practice itself and in working as a reference professional by solving practical problems presented by society and maintaining the trust of lay clients^(7)^. The care provided by nurses in health promotion and prevention actions – particularly in public health – is based on looking at the human being in its entirety, through a contextualized and participatory approach towards users^(20,21)^.

The work of graduates in public health services in their hometowns thus suggests technical authority in public health in the eyes of society, supporting the achievement of Professional Status^(2)^. Furthermore, since professional autonomy is provided by the support and trust of society in the profession, connection with the population proves to enhance public health nurses’ autonomy.

Interestingly, CEESP proved to be the onset for the professional qualification process of its graduates, most of whom sought to take new postgraduate courses after specializing. This result suggests that the knowledge produced and acquired by CEESP not only conferred technical and theoretical authority on public health, but also opened doors for the intellectualization of the graduates, forming a professional corpus qualified to provide care in the Unified Health System.

Freidson’s autonomy-expertise-authority tripod is observed to have been a central component of the training of the specialists. Freidson argues that these three elements interact dynamically to define the status, control, and effectiveness of professions^(7)^. Autonomy allows nurses to make decisions based on judgment, supported by their expertise and guaranteed by their scientific and practical knowledge. It is thus possible to offer higher quality care, adapted to the individual needs of patients and the complex demands of the health environment, which contributes to increasing patient confidence, interprofessional collaboration, and the advancement of the profession.

An application of Freidson’s analysis to the historical phenomenon of this study allows us to support the confirmation of the theoretical assumption presented in the introduction: by seeking expertise, the nurses of CEESP/UFMG promoted the development, organization, and legitimization of public health nursing in the society of Minas Gerais, obtaining professional status by occupying prominent positions and becoming higher education professors^(3)^. This highlights that CEESP was a significant distinguishing experience for professional practice by expanding autonomy through the construction of technical and theoretical skills, deepening expertise by promoting solid scientific knowledge and reinforcing authority by a formal recognition of the title and prominence in the job market^(2)^.

In this context, the findings on the training of nurses with intellectual capital in public health also respond to the objectives of CEESP in providing support for the creation of the Master’s Program in Nursing at EEUFMG, since some of these graduates returned to the School of Nursing to take it and several professors from the course became permanent members of the *stricto sensu* postgraduate program. Thus, CEESP worked as a strategy to pave the way for the implementation of the master’s program in 1994, as the course functioned as a laboratory for approaches to scientific production beyond specialization and is generally considered to provide technical-scientific training for work activity.

The limitations of the study involved the lack of information in the Lattes curriculum and the gaps left in the documents, such as the lack of clarification on the time when and reason why the course was discontinued and the complete list of graduates. However, the relevance of this work lies in its evaluation of the advancements of the profession, highlighting the autonomy, expertise, and authority as proposed by Freidson. The analysis of the trajectories of the graduates contributes to identifying successful professional practices, valuing nursing and evidencing the return on state investment in health courses, further reaffirming their relevance for the Brazilian health system.

## CONCLUSION

The Specialization Course in Public Health at EEUFMG was a milestone in the history of professionalization of nursing in Minas Gerais, as it was the first specialization course in public health offered in this Brazilian state. Based on a sociological analysis, it was possible to trace the professional flow of the course graduates and the individual and collective professionalization process promoted by CEESP, highlighting the expertise of public health nurses.

Furthermore, it is worth highlighting that these social subjects formed an autonomous professional group, and their professional loci demonstrated an elevation of the *status quo*, with a leading role in a movement of intellectual preparation/critical mass for the offering of the first Master’s Course in Nursing in the state of Minas Gerais, at EEUFMG, in 1994. Thus, the outlines of the Specialization Course in Public Health Nursing reaffirm the role of EEUFMG as a vanguard in the constitution of expertise in public health in Minas Gerais and confirm Freidson’s triad autonomy-expertise-authority as foundations for the professionalism of nurses.
