# Supplementary material for: A Freidsonian Analysis of The Professional trajectories of Public Health Nursing Specialists in Minas Gerais, Brazil, 1988–1992
Source: Rev Esc Enferm USP. 2024 Nov 8;58:e20240191. doi: 10.1590/1980-220X-REEUSP-2024-0191en (PMC11548916; doi:10.1590/1980-220X-REEUSP-2024-0191en)
Supplement: Translation

# Translation: Análise freidsoniana das trajetórias profissionais de especialistas em enfermagem de saúde pública mineiros, 1988–1992

**DOI:** 10.1590/1980-220X-REEUSP-2024-0191pt

**Authors:** Isabella Lara Maia de Carvalho, Pacita Geovana Gama de Sousa Aperibense, Alice Gomes Frugoli, Maria Angélica de Almeida Peres, Sagrario Gómez-Cantarino, Fernanda Batista Oliveira Santos

**Affiliations:** 1 Universidade Federal de Minas Gerais, Escola de Enfermagem, Graduação em Enfermagem, Belo Horizonte, MG, Brasil.; 2 Universidade Federal do Rio de Janeiro, Departamento de Enfermagem Fundamental, Macaé, RJ, Brasil.; 3 Universidade Federal de Minas Gerais, Escola de Enfermagem, Programa de Pós-graduação em Enfermagem, Belo Horizonte, MG, Brasil.; 4 Universidade Federal do Rio de Janeiro, Escola de Enfermagem Anna Nery, Departamento de Enfermagem Fundamental, Rio de Janeiro, RJ, Brasil.; 5 Universidad de Castilla-La Mancha, Facultad de Fisioterapia y Enfermería del Campus de Toledo, Departamento de Enfermería, Fisioterapia y Terapia Ocupacional, Toledo, Espanha.; 6 Universidade Federal de Minas Gerais, Escola de Enfermagem, Departamento de Enfermagem Básica, Belo Horizonte, MG, Brasil.

**Keywords:** História da Enfermagem, Enfermagem em Saúde Pública, Educação de Pós-graduação em Enfermagem, Especialização, Ocupações em Saúde

## Abstract

**Objetivo:**

Analisar as contribuições do Curso de Especialização em Enfermagem de Saúde Pública (1988–1992) da Universidade Federal de Minas Gerais para a elevação da *expertise* e do status profissional de seus egressos.

**Método:**

Pesquisa de dimensão histórico-social inserida nos domínios da história da enfermagem e das especialidades. As fontes incluíram 20 fichas de inscrição, documentação de matrícula dos egressos e 3 documentos referentes ao Curso, coletados no Centro de Memória da Escola de Enfermagem da Universidade Federal de Minas Gerais, triangulados com currículos acessados na plataforma Lattes/CNPq e analisados no arcabouço da Sociologia das Profissões, de Eliot Freidson.

**Resultados:**

A habilitação dos especialistas baseou-se na estratégia de construção de saberes direcionados às transformações sociossanitárias. A especialização apoiou o delineamento da carreira profissional dos egressos em cargos administrativos de liderança, gerenciamento e visibilidade na saúde pública, ao passo que o perfil do grupo se apresentou voltado à academia, espaço que forja conhecimento próprio.

**Conclusão:**

A especialização oportunizou exercício da autonomia profissional de seus egressos, elevando o *status quo*, com contribuições para a profissionalização da enfermagem de saúde pública.

## INTRODUÇÃO

O Curso de Especialização em Enfermagem de Saúde Pública (CEESP), ofertado entre 1988 e 1992, foi uma das primeiras iniciativas de pós-graduação em enfermagem do estado de Minas Gerais. Esta ocorreu na Escola de Enfermagem da Universidade Federal de Minas Gerais (EEUFMG), instituição criada em 1933, no bojo das estratégias de investimentos estatais em saúde pública no Brasil^(1)^.

No âmbito dos problemas de saúde pública do início do século XX, o governo brasileiro lançou fortemente a sua atenção à formação qualificada de enfermeiros como estratégia para reformular o modelo de atenção à saúde do país. Assim, a história da enfermagem brasileira está pautada, desde os seus primórdios, na saúde pública e no paradigma da modernidade. Eles caminharam juntos, apoiando a constituição do profissionalismo que a enfermagem detém hoje perante a sociedade e o Estado^(2)^.

Ao longo da segunda metade do século XX, a saúde pública brasileira passou por importantes reconfigurações impulsionadas pela luta por direitos civis. A Reforma Sanitária de 1986 ampliou o conceito de saúde. As modificações necessárias transcenderam os limites de uma reforma administrativa e financeira. A Constituição deu origem ao Sistema Único de Saúde (SUS) e atribuiu ao Ministério da Saúde (MS) o papel de fomentador e incentivador do desenvolvimento científico e tecnológico e da inovação na área da saúde, conforme preconiza o artigo 200 da Constituição Federa^(3,4)^.

Historicamente, as escolas de enfermagem brasileiras se reconfiguraram diante de mudanças do contexto sociopolítico e tais movimentações foram fundamentais para o processo de profissionalização da enfermagem no país. As reformulações do sistema nacional de saúde na década de 1980 refletiram também na formação ofertada pela EEUFMG. Esta foi protagonista de ações extramuros na saúde pública mineira, como a realização de campanhas de vacinação, atuação emergencial em enchentes e prestação de serviços gratuitos de enfermagem em diversos municípios do estado. Foi vanguardista dos processos de profissionalização da enfermagem mineira, implantando um ensino que também valorizava a enfermagem de saúde pública, com importantes contribuições para o cenário de saúde nacional e internaciona^(5,6)^.

Na ótica da Sociologia das Profissões de Eliot Freidson, pensada para a profissão médica, destacam-se dois elementos na profissionalização: a *expertise* e o status profissional. A *expertise* é o conhecimento próprio da profissão que fornece a essência convencional do aprendizado dos *experts* em escolas profissionais, que outras profissões não detêm, sendo cunhada por formação específica submetida a regulamentos formais. O status profissional representa a autoridade técnica e legal na divisão do trabalho de uma ocupação e reflete a posição de importância que a profissão apresenta para a sociedade^(7)^.

No âmbito da análise da enfermagem como campo profissional, o referencial teórico de Freidson ganhou notoriedade nos estudos epistemológicos, especialmente os de cunho histórico(^1,2,6,8,9,10^). Dessa forma, pesquisadoras identificaram os fatores da profissionalização de Freidson no trabalho da enfermagem, concluindo que o enfermeiro é um profissional autônomo e com regulação própria do seu exercício profissiona(^2^>). Ao tomarmos essa análise para a enfermagem, a fim de estabelecer um espaço como campo profissional e área de conhecimento científico que responde às necessidades sociais em saúde, a qualificação profissional e a produção de conhecimento próprio têm se mostrado componentes fundamentais para o enfermeiro^(2,5)^. Acredita-se que este deve se integrar à sociedade e visibilizar a sua trajetória de construção de saberes e práxis e ocupar lócus de interesse para se tornar um profissional valorizado por seus pares e por aqueles que consomem o seu trabalho.

Nessa perspectiva, apesar da existência do CEESP ser reconhecida na literatura, não foram elucidados os desdobramentos dessa formação em saúde pública para os titulados nesse curso de formação em pós-graduação *lato sensu* em enfermagem em Minas Gerais^(6)^. Ademais, a caracterização dos sujeitos envolvidos e as estratégias adotadas na Reforma Sanitária Brasileira ainda são pouco exploradas em estudos sobre este fenômeno. Sendo assim, é preciso visibilizar os empreendimentos históricos da enfermagem pela saúde coletiva brasileira, bem como a formação profissional para assumir tais iniciativas como forma de notabilizar a atuação dessa categoria na área da saúde no cenário naciona(^11,12,13^).

Pressupôs-se que enfermeiros que cursaram o CEESP na UFMG e se dedicaram à construção de uma carreira profissional pautada na construção de conhecimento próprio e específico para essa área ocuparam cargos de destaque, obtendo status profissional. Portanto, este estudo buscou analisar as contribuições do Curso de Especialização em Enfermagem de Saúde Pública (1988–1992) da Universidade Federal de Minas Gerais para a elevação da *expertise* e do status profissional de seus egressos.

## MÉTODO

### Desenho de Estudo

Trata-se de pesquisa no campo da História, de dimensão histórico-social, inserida nos domínios da história das especialidades e da história da enfermagem, com abordagem da história documental/textua^(14,15)^. Foi realizado um estudo de trajetória de egressos, identificando os espaços alcançados pelos titulados após a conclusão do Curso de Especialização em Enfermagem de Saúde Pública.

### Local

O cenário de estudo foi a Escola de Enfermagem da Universidade Federal de Minas Gerais, localizada em Belo Horizonte, capital do estado de Minas Gerais. Para o delineamento temporal, estabeleceu-se como marco inicial o ano de 1988, referente ao ingresso da primeira turma no CEESP, e, como marco final, 1992, ano em que se diplomou a última turma, de acordo com os registros disponíveis.

### Critérios de Seleção

O Centro de Memória da Escola de Enfermagem da UFMG (CEMENF) possui em seu acervo uma coleção documental sobre as especializações em enfermagem ofertadas pela instituição, sendo esta a escolhida para esta pesquisa. O *corpus* documental (população) da coleção do CEESP é constituído por: Plano de Criação do CEESP (1987), Relatório de Atividades 1988–1989 do CEESP (1989), 84 fichas de inscrição de alunos para o curso e documentação de matrícula e identificação dos egressos do CEESP que concluíram o curso nos anos entre 1988 e 1992, incluindo requerimento de matrícula, comprovante de conclusão do curso de graduação, histórico escolar do ensino médio e do ensino superior, *curriculum vitae*, atestado de afastamento e declaração de recomendação, certidão de pessoa física, carteira de identidade, certidão de quitação eleitoral e certificado comprobatório da situação militar.

Os critérios de inclusão das fontes diretas foram: menção ou discurso livre acerca deste curso de especialização, fichas de inscrição e documentação de identificação de egressos do CEESP concluintes entre os anos de 1988 e 1992. Adotaram-se como critérios de exclusão: fichas ilegíveis e/ou danificadas que comprometessem a sua leitura e compreensão. Nesta primeira etapa, foram selecionadas 83 fichas.

Além disso, para compor dados sobre a trajetória profissional de egressos após a conclusão do curso, realizaram-se buscas na Plataforma Lattes, mantida pelo Conselho Nacional de Desenvolvimento Científico e Tecnológico (CNPq). A escolha dessa plataforma justifica-se por ser o principal sistema virtual brasileiro de currículos científicos e ter acesso aberto, permitindo assim a confirmação da identidade dos egressos a partir da triangulação das fontes. Quanto à Plataforma Lattes, incluiu-se o currículo de egressos do CEESP concluintes entre os anos de 1988 e 1992. Foram excluídos os currículos que não dispunham de informação para mapear a trajetória profissional dos egressos, a fim de identificar a influência do curso no alcance da *expertise* e ampliação do status desse grupo. Assim, nessa segunda etapa, vinte currículos compuseram a amostra final.

### Coleta de Dados

A coleta de dados ocorreu entre dezembro de 2023 e janeiro de 2024, no acervo do CEMENF e na Plataforma Lattes, aplicando-se os critérios de inclusão e exclusão. Os documentos foram digitalizados e gravados em plataforma de armazenamento de dados on-line, catalogados, condensados em planilha eletrônica Excel® e, posteriormente, foram disponibilizados pelo museu como coleção documental de domínio público por intermédio do software AtoM® (Access to Memory – um aplicativo de descrição arquivística, baseado nas normas do Conselho Internacional de Arquivos).

Passou-se então à busca dos egressos do CEESP na Plataforma Lattes do Conselho Nacional de Pesquisa. Como a documentação do CEMENF permitiu sua identificação nominal, procedeu-se à procura de cada um deles, colocando-se seus nomes completos no buscador de currículos no sítio eletrônico https://buscatextual.cnpq.br/buscatextual/busca.do?metodo=apresentar.

### Análise e Tratamento dos Dados

Após a heurística ou leitura inicial e recolha de todos os documentos do CEESP, iniciou-se a busca pelos documentos de interesse para a hermenêutica em termos de se saber em que medida as informações fornecidas por estes respondiam às questões inicialmente levantadas. Para tal, procedeu-se às críticas interna e externa documental, verificando-se a validade e a coerência a partir da própria fonte, bem como a sua veracidade quanto aos fatores externos e o contexto sociossanitário dos anos 1980–1990. Uma ficha documental foi utilizada como instrumento para extrair as informações referentes aos egressos do CEESP nessa fase. Ela continha as seguintes questões: “Quais são as contribuições do CEESP (1988–1992) da UFMG para a trajetória de seus egressos? O CEESP elevou a *expertise* e o status profissional de seus egressos?”. Os achados foram então analisados considerando-se o arcabouço teórico da profissionalização em saúde de Eliot Freidson e suas interpretações para o campo profissional da enfermagem^(2,7)^.

### Aspectos Éticos

A pesquisa contou com documentos de acervo histórico público e de plataforma virtual de acesso irrestrito por meio da internet. Além disso, para garantir a privacidade de informação dos egressos e impossibilitar a sua identificação, os dados foram anonimizados, não havendo conflito de interesses e dispensando o parecer de Comitê de Ética, conforme previsto nas Resoluções 466/2012 e 510/2016 do Conselho Nacional de Saúde (CNS).

## RESULTADOS

O documento nomeado como “Plano de Criação do CEESP” (1987) permitiu identificar que o projeto do Curso de Especialização em Enfermagem de Saúde Pública foi idealizado com o intuito de aprofundar conhecimentos próprios da enfermagem e favorecer o desenvolvimento de habilidades profissionais da assistência em nível ambulatorial. O objetivo do curso era capacitar enfermeiros para a prestação de serviços e para o estudo sistematizado do trabalho de enfermagem na atenção primária. Além disso, como documento principal de criação do CEESP, o registro propõe que esse curso de especialização deveria servir como suporte e preparação para a criação do Programa de Mestrado em Enfermagem na área de Saúde Pública. O mestrado em enfermagem foi criado na EEUFMG em 1994, com forte engajamento de professores enfermeiros da saúde pública^(5)^.

O “Relatório de Atividades 1988–1989” (1989) do CEESP descreveu o desenvolvimento do curso dentro de um processo pátrio da capacitação de docentes iniciado por Escolas de Enfermagem de três universidades brasileiras: Universidade Federal da Bahia, Universidade de São Paulo e Universidade Federal de Minas Gerais. Vale ressaltar, portanto, que o CEESP foi o primeiro curso a ser implantado a partir de uma decisão nacional de especializar enfermeiros da atenção primária, naquele momento denominada “atenção básica à saúde” pelo Movimento Sanitário Brasileiro^(16)^, com vistas à consolidação da Reforma Sanitária. Foi promovido, por meio de convênios e acordos, com o apoio técnico e financeiro do Instituto Nacional de Assistência Médica da Previdência Social (INAMPS), Organização Pan-Americana de Saúde (OPAS), Ministério da Saúde, Secretaria de Estado da Saúde de Minas Gerais (SES/MG) e Secretaria Municipal de Saúde de Belo Horizonte (SMS/BH). Assim, a EEUFMG incumbiu-se não somente da função de qualificar enfermeiros inseridos no processo de trabalho da atenção primária, como também do papel de formar centros de referência para as demais Escolas de Enfermagem do país por meio da educação pública e totalmente gratuita, honrando sua responsabilidade ao retornar para a própria sociedade o investimento governamental recebido.

A estrutura curricular do curso dispunha de disciplinas que abordavam a capacitação do enfermeiro para a função supervisor-instrutor, o controle de doenças transmissíveis, saúde da mulher e da criança, saúde do homem urbano e rural, capacitação pedagógica e administração de serviços e processos metodológicos. A carga horária total era de 960 horas distribuídas em dois semestres letivos da Universidade, sendo as atividades práticas desempenhadas em quatro Centros de Saúde de Belo Horizonte definidos como campos fixos para todas as disciplinas, e em campos móveis definidos a partir da natureza e especificidade de cada uma. Para fins de comparação, nas décadas de 1980 e 1990, no Brasil, a carga horária dos cursos de especialização variava de acordo com os centros educacionais e a conformação das diretrizes de cada um. Entretanto, a partir da Resolução CNE/CES nº 3 de 1999, a carga horária mínima dos cursos de pós-graduação *lato sensu* passou a ser de 360 horas^(17)^, o que denota o rigor acadêmico e a profundidade da formação oferecida pelo CEESP, preparando profissionais diferenciados para o mercado de trabalho.

A matrícula no curso era realizada mediante apresentação da ficha de inscrição devidamente preenchida, comprovante de conclusão do curso superior de Enfermagem e Obstetrícia, histórico escolar, *curriculum vitae*, comprovação de estar em dia com as obrigações militares e eleitorais e declaração do órgão empregador sobre a importância do curso para o trabalho do candidato e a disposição da sua liberação para realizar o curso. O processo seletivo se deu por ocasião do acordo entre as instituições que apoiaram o curso, sendo priorizados os enfermeiros indicados por elas, com a seguinte distribuição das trinta vagas a cada semestre do curso: dez vagas para enfermeiros indicados pelo INAMPS; dez vagas para enfermeiros indicados pela SES/MG; cinco vagas para enfermeiros indicados pela SMS/BH; cinco vagas para enfermeiros selecionados pela EEUFMG, com prioridade para docentes do curso de graduação em enfermagem. Eventuais vagas remanescentes seriam destinadas a enfermeiros em geral e, se o número de candidatos excedesse o número de vagas, seriam conduzidas entrevistas e análises do currículo por uma banca examinadora constituída para esse fim.

Segundo os documentos acessados, sabe-se que, no período de 1988 a 1992, pelo menos 83 alunos foram titulados especialistas em enfermagem de saúde pública pelo curso. No entanto, o acesso às fontes diretas do CEMENF permitiu a caracterização da trajetória profissional de vinte egressos do CEESP a partir das fichas de admissão/identificação e dos currículos Lattes. Dentre os egressos, observou-se a predominância de mulheres (95%), com faixa etária média de 27 anos e autodeclaradas brancas (Figura 1). É importante destacar a presença de uma egressa negra e dois egressos do sexo masculino. Nota-se a predominância da formação de enfermeiros naturais de Minas Gerais, provenientes de quinze municípios mineiros. Ademais, enfatiza-se a formação de egressos do Rio de Janeiro e Rio Grande do Sul.

**Figura 1:** Frequência de raça/cor, sexo, naturalidade e instituições de formação superior dos egressos.

Quanto à diplomação, cabe destacar que a maior parte se formou em universidades mineiras, localizadas nas cidades de Belo Horizonte, Juiz de Fora, Alfenas e Itajubá, sendo as mais frequentes a UFMG, Pontifícia Universidade Católica de Minas Gerais (PUC-MG) e Universidade Federal de Juiz de Fora (UFJF) (Figura 1).

As fontes evidenciaram ainda que quase a totalidade dos enfermeiros que ingressaram no curso tinha histórico de trabalho no setor público de saúde. Dentre a documentação de matrícula apresentada pelos egressos para se inscreverem no curso, as declarações do órgão empregador serviram como cartas de recomendação para atestar a relevância do curso na formação profissional dos referenciados, redigidas por superintendentes dos cargos previamente ocupados.

As onze recomendações submetidas, acessíveis no acervo do CEMENF, são da autoria de órgãos pertencentes ao Estado (Figura 2). Ressalta-se o apoio administrativo das unidades da rede estadual, em que alguns desses egressos trabalhavam, para a qualificação profissional, destacando a importância dessa atividade para a “melhoria da assistência prestada à população” e para a atividade de gestão.

**Figura 2:** Declarações de recomendação dos egressos. Fonte: Documentação de matrícula do Curso de Especialização em Enfermagem de Saúde Pública 1989–1990, acervo do Centro de Memória da Escola de Enfermagem da Universidade Federal de Minas Gerais.

Apesar da indicação para a realização desse curso, cabe destacar que os candidatos tinham formação em outros cursos de pós-graduação, a saber: Aperfeiçoamento em Gerência Hospitalar, Gerência de Recursos Humanos, Gerência de Unidades Hospitalares, Fortalecimento das Ações de Imunização nos Territórios Municipais, Serviços de Gestão e Administração do Processo de trabalho em Enfermagem, Habilitação em Enfermagem de Saúde Pública e Especialização em Saúde Pública à parte do CEESP/UFMG. Outrossim, seis dos vinte egressos tinham a média de dois ou mais cursos de pós-graduação dentre o grupo.

A análise dos currículos dos egressos aponta que, no período entre seis meses e um ano do término do CEESP, esses profissionais se destacaram em atuações no setor público. Tanto entre aqueles que permaneceram no estado de Minas Gerais (quatorze dos vinte egressos) quanto entre os que regressaram para suas respectivas cidades natais para colaborar com o cenário de saúde local (seis dos vinte egressos).

Acerca de cargos exercidos consecutivamente ao curso, todos os vinte egressos analisados seguiram como servidores públicos e exerceram funções em diferentes instituições de serviços de saúde, dentre elas Secretarias Municipais de Saúde e Secretaria Estadual de Saúde de Minas Gerais, Centros de Saúde, hospitais, policlínicas, ambulatórios, Postos de Atendimento Médico, Unidades Básicas de Saúde, Coordenadorias Regionais de Saúde e universidades públicas mineiras em cursos de graduação e pós-graduação em Enfermagem.

As posições preenchidas pelos egressos no setor público que mais se destacam após cursarem a Especialização foram: Gerência de Unidade Básica de Saúde, Direção e Administração do setor ambulatorial do Hospital das Clínicas da UFMG, Supervisão do Serviço de Enfermagem, Direção de Regional Metropolitana de Saúde, Coordenação e Supervisão de Multivacinação, Direção do Departamento de Enfermagem Materno-Infantil e Saúde Pública da Escola de Enfermagem da UFMG, Especialista em Política de Gestão de Saúde, Instrutor supervisor e Professor da EEUFMG. Ainda, dentre esses egressos, dez operaram no âmbito público por duplas jornadas de trabalho, sendo dois deles mediante migração pendular entre Belo Horizonte e municípios da região metropolitana.

O tipo de atividade desenvolvida variou entre as seguintes dimensões do processo de trabalho do enfermeiro: assistência (20%), gerência (37%), pesquisa (3%) e educação (40%). Somente um egresso exerceu funções no setor privado ao lecionar em uma instituição particular de ensino superior de enfermagem de Belo Horizonte. No que tange ao avanço de suas qualificações profissionais pós-CEESP, 95% possuíam mais de uma titulação e realizaram pelo menos um tipo dentre os três cursos Especialização (33), Mestrado (13) ou Doutorado (6).

Do grupo analisado, 16 egressos tornaram-se especialistas nos seus respectivos objetos de estudo do Mestrado e/ou Doutorado: Gerência de Unidades Básicas de Saúde, Planejamento e Administração de Sistemas de Saúde, Enfermagem do Trabalho, Ergonomia, Urgência e Atendimento Pré-hospitalar Móvel, Saúde da Família, Educação Profissional na Área da Saúde, Gestão Microrregional de Saúde, Docência do Ensino Superior, Ativação de Processos de Mudança, Estomaterapia, Enfermagem de Saúde Pública para o Sistema Único de Saúde, Vigilância Sanitária, Planejamento e Gestão Estratégica em Saúde, Enfermagem Gerontológica e Geriátrica, Epidemiologia em Serviços de Saúde, Micropolítica da Gestão e Trabalho em Saúde e Epidemiologia em Saúde do Trabalhador.

Doze desses egressos foram titulados Mestres na Grande área de Ciências da Saúde e na Área de Enfermagem. A Subárea variou entre Enfermagem em Saúde Coletiva, Epidemiologia, Saúde da Família e Atenção Básica, Saúde e Serviços Sociais. A Especialidade abrangeu Saúde Pública, Cuidado à Saúde das Populações Humanas, Meio Ambiente e Sustentabilidade, Política Econômica e Administração Pública em Geral, Vulnerabilidade Social, Saúde da Mulher e do Recém-nascido, Planejamento em Saúde e Avaliação do Sistema de Informações. Um egresso tornou-se Mestre na Grande área de Ciências Sociais Aplicadas, na Área de Administração e Subárea de Administração Pública, na Especialidade Defesa e Seguridade Social. Dez dos doze egressos realizaram outros cursos de Especialização previamente ao curso de Mestrado.

Dos citados acima, cinco egressos se diplomaram Doutores na Grande área de Ciências da Saúde e na Área de Enfermagem. A Subárea constitui-se de Saúde Coletiva e Enfermagem de Doenças Contagiosas e a Especialidade comportou Saúde da Família e Atenção Básica, Processo de Organização em Serviços de Saúde, Doença de Chagas, Infecções Sexualmente Transmissíveis na Adolescência e Serviços Coletivos Prestados pela Administração Pública na Esfera da Seguridade Social. Um egresso se diplomou como Doutor na Grande área de Ciências Biológicas, Subárea de Parasitologia-Epidemiologia e Especialidade Dengue. Todos esses seis egressos realizaram curso de Mestrado antes de iniciar o Doutorado.

Quanto aos cargos atuais ou os últimos de que se tem registro dentre os ocupados pelos egressos do curso, 18 são de servidores públicos estatutários ou celetistas, vinculados aos seguintes serviços de saúde: Secretarias Municipais de Saúde e Secretaria de Estado de Saúde de Minas Gerais, hospitais, Coordenadorias Regionais de Saúde, Departamento de Atenção à Saúde do Trabalhador, Fundação Hospitalar do Estado de Minas Gerais, Ministério da Saúde, Serviço de Atendimento Móvel de Urgência (SAMU), Centro de Referência em Saúde do Trabalhador e universidades do Sudeste do Brasil em cursos de graduação e pós-graduação em Enfermagem e Medicina.

No que diz respeito aos níveis dos cargos ocupados pelos egressos do CEESP, destacam-se posições elevadas, incluindo Professor e pesquisador de Enfermagem na UFJF e UFMG, Coordenador de Enfermagem no Hospital das Clínicas da UFMG, Coordenador de Epidemiologia, Diretor de Vigilância em Saúde, Secretário de Vigilância Epidemiológica, Coordenador geral do SAMU e Superintendente de Gestão do Trabalho. Além disso, foram identificadas conexões voluntárias com a Associação Brasileira de Enfermagem (ABEn Nacional) e a participação no Corpo Editorial da Revista Mineira de Enfermagem (REME).

O tipo de atividade desenvolvida variou entre as quatro dimensões do trabalho do enfermeiro: assistencial (3,7%), gerencial (37,04%), pesquisa (18,52%) e ensino (40,74%). Cinco egressos desempenharam jornadas duplas de trabalho, sendo que dois seguiram em atividades de docência e pesquisa no setor privado. A atuação destes se deu em universidades mineiras em cursos de graduação e pós-graduação em Enfermagem e Medicina. Os postos preenchidos foram Professor e Pesquisador e Coordenador do Curso de Enfermagem.

## DISCUSSÃO

A estrutura curricular descrita no projeto para a criação do curso explicita a preocupação em transmitir o conhecimento em saúde pública, com a finalidade de resgatar a competência técnica, forjar os saberes próprios da profissão e habilitar profissionais de referência para a Reforma Sanitária^(6)^. Além disso, com a implementação dos princípios do SUS e da atenção primária à saúde, fez-se necessária a qualificação de enfermeiros aptos a intervir política e administrativamente no processo de reorganização dos serviços locais de saúde^(18)^, o que nos leva a inferir que as disciplinas de capacitação pedagógica e administração de serviços e processos metodológicos compunham o currículo com vistas a desenvolver essas habilidades e competências.

A respeito do perfil socioeconômico dos egressos, nota-se a influência histórica difundida socialmente^(19)^, perpetuado por uma identidade profissional fortemente relacionada com a questão religiosa e do próprio padrão Nightingale, determinando o ingresso majoritário de mulheres brancas jovens nos cursos de enfermagem superior. Entretanto, destaca-se a titulação de especialista a uma mulher negra e dois homens pelo CEESP. A presença de uma mulher negra e dois homens no CEESP reafirma o movimento de superação de paradigmas elitistas de formação e de ruptura com a segregação e desigualdade nas relações de trabalho promovido pela EEUFMG desde a sua criação. Cabe destacar que a escola foi pioneira na formação de uma enfermeira negra na sua primeira década de funcionamento^(1)^.

A análise da trajetória profissional de egressos permitiu delimitar um fluxo profissional (Figura 3). Sendo a maioria natural do próprio estado, a formação acadêmica dos egressos ocorreu na capital; ao concluírem-na, retornaram às suas respectivas cidades natais e, após a conclusão da pós-graduação, com o título de especialista, assumiram cargos de liderança em saúde pública em seus municípios de origem e/ou tornaram-se docentes em enfermagem, diferentemente da realidade histórica dos primórdios do processo de especialização de profissionais enfermeiras no estado. Cabe ressaltar que o fluxo da Figura 3 se refere aos egressos do CEESP caracterizados na Figura 1.

**Figura 3:** Fluxo profissional dos egressos do Curso de Especialização em Enfermagem de Saúde Pública.

Sabe-se que as primeiras professoras tituladas especialistas, mestres e doutores em Minas Gerais precisaram se qualificar fora do estado e do Brasil. Entretanto, após a conclusão dessas titulações, retornaram para a EEUFMG para formar novos enfermeiros e propulsar a criação de cursos de especializações no estado, bem como liderar ações de profissionalização da enfermagem mineira e naciona(^1,5,6^).

Desse modo, destaca-se um ciclo de profissionalização (Figura 4) que, ao ocorrer na enfermagem mineira, reflete um arquétipo produzido pelas primeiras enfermeiras formadas na Escola de Enfermagem Anna Nery, apontando para o compromisso social que esses pioneiros tinham com seus espaços de formação e atuação^(5)^. Além disso, depreende-se que os cidadãos naturais de municípios mineiros, com desafios de saúde pública, buscaram se profissionalizar e adquirir *expertise* ^(7)^ nessa área individualmente. Uma vez especializados, passaram a liderar os serviços de saúde pública locais, capacitando os profissionais que ali atuavam, motivando-os a se especializar, como demonstra a Figura 4.

**Figura 4:** Ciclo da profissionalização da Enfermagem de Saúde Pública em Minas Gerais no final da década de 1990.

Observa-se que o campo de profissionalização obedece a uma tendência de conformação em que o próprio Curso vai sendo tensionado pelo movimento de profissionalização e atendimento de demandas (1 e 2 da Figura 4), incorporando as necessidades sociossanitárias como instrumentos de transformação de si (3 da Figura 4), do campo em que atua (4 da Figura 4) e dos sujeitos envolvidos (5 da Figura 4). Estes, por sua vez, também passam a se recolocar nesse campo (6 da Figura 4) em pontos diferentes e causando impactos em seus trabalhos (3’ da Figura 4), que serão singulares (4’ da Figura 4) e afinados às novas necessidades sociais e, assim, sucessivamente, os profissionais com a *expertise* vão influenciando outros profissionais, inclusive os recém-formados, mantendo a espiral do conhecimento em constante desenvolvimento progressivo (٣’’ e [...] da Figura ٤).

Em relação ao processo de profissionalização do enfermeiro em saúde pública em Minas Gerais, enfatiza-se que 55% das titulações de especialista pelo CEESP foram apoiadas diretamente pelos serviços de saúde empregadores dos inscritos, por meio de carta de recomendação. Isso demonstra que o CEESP detinha valor social perante o Estado, que investiu na qualificação dos enfermeiros como a profissão que detém expertise em saúde pública, protegendo assim seu espaço de autonomia e corpo de conhecimento nessa área^(2,7)^. Portanto, nota-se o credencialismo do trabalho do enfermeiro na saúde pública em MG.

É importante, ainda, notar que os próprios enfermeiros, após titulados, assumiram papel de docência e/ou liderança, fomentando a capacitação nos serviços que os recomendaram, apontando para o domínio do seu corpo de conhecimento^(7)^ e escopo de atuação em saúde pública e apoiando o curso de interiorização da profissionalização da enfermagem no estado de Minas Gerais. Ademais, a expertise do enfermeiro de saúde pública formado pelo CEESP foi evidenciada a partir da constatação dos requisitos teóricos da formação profissional postulada por Freidson^(7)^, sendo eles o aprendizado de conhecimentos específicos por formação em escola profissional própria submetida a regulamentos formais – nesse caso, a EEUFMG.

Ainda, segundo Freidson, a *expertise* expressa-se no próprio exercício profissional e na atuação como profissional de referência, ao solucionar problemas práticos apresentados pela sociedade e sustentar-se na confiança da clientela leiga^(7)^. Especialmente na saúde pública, o cuidado prestado pelo enfermeiro nas ações de promoção e prevenção da saúde se dá a partir do olhar para o ser em sua integralidade, por meio de uma abordagem contextualizada e participativa do usuário^(20,21)^.

Desse modo, o trabalho dos egressos nos serviços de saúde pública em seus contextos natais aponta para a posição de autoridade técnica em saúde pública diante da sociedade, corroborando para a conquista do Status Profissiona^(2)^. Além disso, uma vez que a autonomia do profissional se dá pelo apoio e confiança da sociedade na profissão, a atuação de enfermeiros em um contexto de vínculo com a população demonstra-se como um fator potencializador da autonomia profissional do enfermeiro de saúde pública.

Um achado interessante é que o CEESP se demonstrou um deflagrador do processo de qualificação profissional dos egressos, que, em sua maioria, buscaram a realização de novos cursos de pós-graduação após se especializarem. Esse resultado sugere que o conhecimento produzido e adquirido pelo CEESP não somente conferiu autoridade técnica e teórica sobre saúde pública, mas abriu portas para a intelectualização dos titulados, formando um corpus profissional qualificado para o atendimento no sistema único de saúde.

Na concepção freidsoniana, observa-se o tripé autonomia-*expertise*-crendencialismo como componente central da formação dos especialistas. Freidson argumenta que esses três elementos interagem dinamicamente para definir o status, o controle e a eficácia das profissões^(7)^. A autonomia permite que enfermeiros tomem decisões baseadas em julgamento, sustentado por sua *expertise*, garantida pelo conhecimento científico e prático adquiridos. Deste modo, é possível oferecer cuidados de maior qualidade, adaptados às necessidades individuais dos pacientes e às demandas complexas do ambiente de saúde, o que contribui para aumentar a confiança dos pacientes, a colaboração interprofissional e o avanço da profissão.

A análise freidsoniana, aplicada ao fenômeno histórico deste estudo, nos permite sustentar a confirmação do pressuposto teórico apresentado na introdução de que, ao buscarem *expertise*, os enfermeiros do CEESP/UFMG promoveram o desenvolvimento, a organização e a legitimação da enfermagem de saúde pública na sociedade mineira, obtendo status profissional ao ocuparem cargos de destaque e se tornarem docentes do ensino superior^(3)^. Isso evidencia o CEESP como um diferencial significativo para a prática profissional ao ampliar a autonomia por meio da construção de competências técnicas e teóricas, aprofundamento da *expertise* pela promoção de conhecimento científico sólido e reforço do credencialismo pela obtenção de reconhecimento formal do título e destaque no mercado de trabalho^(2)^.

Nesse contexto, os achados de preparo de enfermeiros com capital intelectual em saúde pública respondem também aos objetivos do CEESP em fornecer suporte para a criação do curso de mestrado em enfermagem na EEUFMG, uma vez que parte desses egressos retornou à Escola de Enfermagem para cursá-lo e vários docentes do curso tornaram-se membros permanentes do programa de pós-graduação *stricto sensu*. Assim, o CEESP funcionou como estratégia que abriu frentes para a implementação do mestrado em 1994, na medida em que o curso funcionou como um laboratório de aproximações com a produção de ciência, para além da especialidade, que é comumente vista como uma capacitação técnico-científica para a atividade laboral.

Aponta-se que as limitações do estudo envolveram a falta de informações nos currículos Lattes e as lacunas deixadas nos documentos, como a ausência de esclarecimento sobre o motivo e o momento do término da oferta do curso e a relação de titulados no total. No entanto, este trabalho se faz relevante por avaliar os avanços da profissão, destacando a autonomia, *expertise* e credencialismo propostos por Freidson. A análise da trajetória dos egressos contribui para identificar práticas profissionais bem-sucedidas, valorizando a enfermagem e evidenciando o retorno do investimento estatal nos cursos de saúde, reafirmando ainda mais a sua relevância para o funcionamento do sistema de saúde brasileiro.

## CONCLUSÃO

A oferta do Curso de Especialização em Saúde Pública pela EEUFMG é um marco na história da profissionalização da enfermagem mineira por ser o primeiro curso de especialização em saúde pública ofertado no estado. A partir da análise sociológica, foi possível traçar o fluxo profissional dos egressos do curso e o processo de profissionalização individual e coletivo promovido pelo CEESP, evidenciando a *expertise* do enfermeiro de saúde pública.

Ademais, vale destacar que esses sujeitos sociais formaram um grupo profissional autônomo e os lócus profissionais destes demonstraram elevação do *status quo*, com protagonismo de um movimento de preparação intelectual/massa crítica para a oferta do primeiro Curso de Mestrado em Enfermagem do estado de Minas Gerais, pela EEUFMG, em 1994. Assim, os delineamentos do Curso de Especialização em Enfermagem de Saúde Pública reafirmam o papel da EEUFMG como vanguardista na constituição de *expertise* na saúde pública mineira e confirmam a tríade freidsoniana da autonomia-*expertise*-credencialismo como aspectos sustentadores do profissionalismo do enfermeiro.
